# Supplementary material for: Are carbon emissions trading and green financial instruments synergistic? -Comprehensive quantitative research based on content analysis
Source: PLoS One. 2024 Mar 7;19(3):e0298601. doi: 10.1371/journal.pone.0298601 (PMC10919601; doi:10.1371/journal.pone.0298601)
Supplement: S1 Data — (ZIP) [file pone.0298601.s001.zip › Data/地级市Z_F工作报告.zip!/╜¡╬≈/╝¬░▓╩╨/└┤╫╘╣1⁄2╓┌║┼í╛┬φ┐╦╩2╛▌═°í┐_2010.docx]

各位代表：

　　现在，我代表市人民政府，向大会作工作报告，请予审议，并请各位政协委员和列席会议的同志提出意见。

　　一、2009年工作回顾

　　2009年是经济发展最为困难的一年，也是全市上下主动作为、化危为机、逆境突破的一年。面对金融危机的严峻挑战，在市委的坚强领导下，我们以胡锦涛总书记视察吉安为强大动力，认真贯彻落实“三保一弘扬”总体要求，深入实施“主攻项目、决战两区”战略，圆满完成了市二届人大五次会议提出的目标任务。这一年，全市经济实现逆势上扬。全年实现地区生产总值584.11亿元，增长13.7%；财政总收入64.36亿元，增长20.3%，财政支出128亿元，增长27.7%，税收占财政总收入的比重上升至80.5%；固定资产投资589亿元，增长43.4%；财政总收入和地方财政收入、规模工业主营收入和实现利税、固定资产投资、外贸出口、城镇居民人均可支配收入和农民人均纯收入等主要经济指标均提前完成“十一五”规划目标。这一年，一大批打基础、增后劲的项目落地开工，尤其是全市人民期盼已久的华能井冈山电厂二期工程建成发电，总装机容量达到192万千瓦；衡茶吉铁路、峡江水利枢纽、石虎塘航电枢纽、泉南高速吉安段全面开工，发展后劲不断增强。这一年，城市面貌发生显著变化，中心城区井冈山大道二期工程建成通车，“一城三区、两江三岸”的城市框架业已形成，一批完善城市功能、提升城市品质的重点城建项目相继开工。创建国家园林城市通过考查有　　 望获批，创建省级卫生城市通过复核，创建省级文明城市工作先进城市有望获得。这一年，全市群众得到更多实惠。市县两级投入民生资金达26.6亿元，增长34.3%，省政府下达的100个民生指标全面完成。888个新农村建设试点村和20个综合示范点建设任务均已完成，村容村貌发生明显变化。城镇居民人均可支配收入14095元，增长9.5%；农民人均纯收入5018元，增长8.2%。

　　一年来，我们主要抓了以下工作：

　　（一）有效应对危机挑战，经济增长保持良好势头。针对金融危机带来的严重冲击，我们超前谋划，积极应对，创优投资环境，鼓励投资创业，引导产业发展。全力开展企业帮扶，市县组成172个工作组，帮助企业解决生产经营难题，共为园区企业招工5.26万人，免费培训员工6.5万人，落实融资40亿元，企业家信心指数、企业景气指数和工业用电量逐季提升。全市电网最高负荷达55.1万千瓦，工业用电量增长10.6%；主营收入过亿元的企业达145户，比 上年新增27户，经济效益综合指数达到274%，再创历史新高。全力主攻重大项目，各地、各部门抢抓国家政策机遇，主动积极争资　　争项，争取中央和省项目资金17.77亿元，占全省总份额的13.7%。市重点调度的155项重点工程加快推进，成为拉动经济发展的强力“引擎”。全力扩大对外开放，先后组织两轮“百日招商”，实施项目推进“双百会战”，工业、城建、农业及旅游招商均有收获，井开区与深圳南山、泰和与浙江温岭、新干与广东花都、永新与台湾彰化对接出现新气象。全市实际引进内资129.8亿元，增长22.4%，引进亿元以上工业项目82个，进资增幅列全省第一；实际利用外资3.45亿美元，增长16.7%，先后有8家外资企业追加投资3000万美元；外贸出口4.42亿美元，增长65.2%。

　　（二）扎实推进决战“两区”，新型工业化和新型城镇化互动并进。园区建设方面，吉泰工业走廊建设取得实质进展，105国道“二改一”全面开工，吉安至厦门、吉安至深圳实现海铁联运，无水港建设加快推进，电镀集控中心落户开工，省级电子信息产业基地、进出口机械电子产品安全检测中心先后　　 挂牌，服务配套功能不断完善。全市工业园区在“逆水”中前行，井开区被列为国家科技兴贸创新基地和国家风电设备产业化基地，进入全省一类园区，吉安、泰和两县工业园区进入全省二类园区；全市园区基础设施再投入40亿元，标准厂房开建近100万平方米；中泰电子一期、红板一期等重大工业项目建成投产，新干120万吨联碱、永丰400万吨旋窑水泥等项目建设进展顺利；园区完成工业增加值180亿元，上交税金27亿元，分别增长20.8%和21.6%；产业集优集群发展势头强劲，电子信息和医药化工两大产业主营收入迈上百亿元台阶，冶金机械产业主营收入超过200亿元。城区建设方面，我们抓住规划、建设、经营、管理等关键环节，城市总体规划和市域城镇体系规划完成修编报批。中心城区商业网点、公路交通、中小学教育布局等一批专项规划完成编制，近期建设区段控制性详规全覆盖。乡镇总体规划和2517个行政村规划编制全面完成。全市启动实施城建重点工程211项，完成投资45.9亿元，其中中心城区完成投资20亿元，项目数量之多、投资力度之大创历史之最。新禾埠大桥、石阳路改造、正气广场、井冈山大道及阳明路街景整治等工程全面完工，市文化艺术中心、市民中心公园、庐陵文化生态公园等项目加快推进，中心城区新增园林绿地200万平方米，全市城镇化率比上年提升2个百分点。吉安县有望获批国家园林县城，吉水、泰和、安福争创省级园林城市通过考察有望获得，安福、吉水获批省级卫生城市。吉安市和吉安县、吉水县被评为全省推进新型城镇化和城市建设工作先进市县。全省新型城镇化和城市建设现场会在我市成功召开。

　　（三）不断夯实“三农”基础，农业经济持续发展。按照统筹城乡发展的要求，我们坚持以新农村建设和产业化经营为抓手，全面落实惠农政策，“三农”工作创特色、出亮点。农业经济稳步增长，全市粮食总产373.3万吨，增长3.7%，创历史新高；全年生猪出栏334万头，肉牛出栏40万头，家禽出笼6100万羽，分别增长10%、9%和9%；蔬菜生产总面积129万亩，增长3.4%；特种水产品产量5.2万吨，增长10%，农业生产走上规模化、专业化轨道。农业产业化成效明显，新干正邦和金佳谷物、吉州思倍得生猪屠宰、泰和和泰肉牛加工、吉水金田米业、遂川新大地茶油等一批农业龙头项目建成投产，全市农业龙头企业达到202家，其中国家级1家、省级38家；全市新增农民专业合作社438家，累计达到775家；创“中国名牌产品”、“中国驰名商标”各1个，国家“绿标”116个。农业产业化经营形成了规模扩大、领域延伸、链条拉长的新格局。新农村建设顺利推进，全市筹资1.61亿元，完成改路1755公里、改水3.34万户、改厕3.13万户，试点村生活垃圾处理率达80%；农村公路及渡改桥工作走在全省前列；油茶、楠木、茶叶等新兴富民产业加快发展，吉安、遂川、安福、永丰、峡江、新干6县列为全国现代油茶建设示范县。全市随处可见规划有序、功能完善、环境优美、出行方便、富于特色的农家新村。农业综合生产能力得到提升，中央规划内32座病险水库除险加固完成年度任务，改造中低产田13.75万亩，解决25.57万人安全饮水问题。造林绿化“一大四小”工程建设加快推进，全市投资11.8亿元，再造林137万亩，被评为全省第一名，吉州区和吉水、泰和、吉安县被评为全省先进县。全省造林绿化“一大四小”现场会、农村公路暨渡改桥现场会、病险水库除险加固现场会、民族地区“一村一品”现场会先后在我市成功召开。

　　（四）着力培植增长亮点，经济结构进一步优化。坚持把调优结构作为保增长、促发展的根本途径，大力发展现代服务业，努力培植新的经济增长点。加快发展旅游产业，积极整合全市旅游资源，加快景区景点建设，认真调查研究，出台了《关于进一步加快旅游产业发展建设旅游大市的若干意见》，先后在北京、上海、香港等地成功举办了系列旅游推介，以井冈山为龙头的红色旅游品牌进一步打响，全市接待游客1236万人次，旅游总收入74.1亿元，井冈山门票收入突破1亿元，“红、绿、古”三色辉映的旅游发展态势进一步形成。积极培育商贸物流业，安排启动和规划准备一批市场项目，城南钢材市场、工程机械市场开工建设，广场商城、农产品物流中心、城南商业街区等相继推出招商或规划，城市商贸功能布局得到加强；新干箱包市场竣工，有望培育成全国区域生产集散基地。进一步扶持物流企业做强做大，全市货运物流业税收增长65.4%。井冈山机场年旅客发送量达10.6万人次。狠抓房地产业健康发展，年初出台《支持房地产业健康发展意见》，促进房地产市场逐步回暖转旺，全市房地产开发投资增长14.1%；保障性住房建设走在全省前列，全市建成经济适用房2010套、廉租房5020套，改造农村危房3080户，一大批中低收入家庭喜迁新居。努力扩张农村消费需求，加快推进“万村千乡”市场工程，扎实开展农机、家电、汽车下乡，农村消费增长18.8%。全年实现社会消费品零售总额171亿元，增长19%。

　　（五）努力推进改革创新，有效破解发展难题。把保增长与促改革、添活力有机结合起来，先后赴西安、延安、合肥、柳州、长治、长沙、湘潭、萍乡、宜春等地学习考察，进一步解放思想，创新发展举措。积极推进园区体制创新，对井开区外部授权、内部明责，形成区内的事区内办、封闭式管理、开放式运行的新管理体制；按照多予少取、放权放活原则，理顺了吉州、青原工业园区管理体制，促进井开区和两区工业园发展焕发新的生机与活力。大力推进金融学习创新，针对项目建设的资金瓶颈，加快金融创新，组建了市金融工作办公室和七大政府投融资公司，市城投公司在不到6个月内成功发行15亿元城投债券，新庐陵公司为各县市区提供融资贷款13.4亿元，市担保公司为94家企业融资担保4.81亿元。吉安县、泰和县、井冈山市成功引进村镇银行和商业银行驻点。全市新增融资135.5亿元，比上年增长226%，直接用于城镇建设资金达54.3亿元。顺利推进国企改革，全面完成99户国有工业企业改制，安置职工53742人，化解金融机构不良资产32.05亿元，提前5个月完成6户列入省考核的国有工业企业改制工作。稳妥推进征地拆迁，在广大市民的大力支持下，全年新征建设用地8627亩，清理闲置土地1282亩；完成拆迁60.27万平方米，其中中心城区拆迁42万平方米，建成安置房10.1万平方米，确保了重点项目顺利实施。此外，全市事业单位岗位设置全部到位，中心城区环卫体制改革出台并加快推进。

　　（六）深入实施民生工程，积极为民办好实事。坚持把民生工程摆在政府工作的重中之重，着力解决了一批关涉群众切身利益的热点难点问题。年初确定的“10件为民实事”全面完成，中心城区建成经适房9.3万平方米、廉租房3.25万平方米，井冈山大道二期全线通车，市图书馆主体工程竣工，100条背街小巷改造全部完工，竹笋巷农贸市场、青原农贸综合市场开工建设，12个县市区污水处理设施全部建成，完成通村水泥路1537公里，建成140个试点村垃圾处理工程，完成525个20户以上已通电自然村广播电视“村村通”工程，完成移民扶贫搬迁13799人。大力扶持就业再就业，广泛开展了“农民工返乡就业创业服务月”等系列活动，全市城镇新增就业7.09万人，城镇登记失业率3.14%；新增转移农村劳动力9.28万人。狠抓社保扩面征缴，城镇职工养老保险参保人数47.88万人，农村社会养老保险1.24万人，城镇职工基本医疗保险参保人数60万人，城镇居民基本医疗保险、新型农村合作医疗保险实现全覆盖。新农合资金使用率达到96.2%。加大弱势群体救助力度，重点提高城乡低保对象、农村五保、城镇“三无”人员、精减退职老职工等四类困难群体的补助标准，全面开展城乡　　低保整治，全市共清退农村低保对象“人情保”5376户、1.23万人，新增保障对象5519户、1.22万人。扎实推进生态建养和环境保护，井冈山、遂川获得省级生态县称号，全市化学需氧量减排2508吨，二氧化硫减排6850吨，中心城区环境空气达到二级标准以上，赣江吉安段水质达到国家Ⅲ类标准以上，饮用水源水质达标率100%，显现出“天更蓝、山更绿、水更清”的如画景象。

　　（七）统筹发展各项事业，社会保持和谐稳定。教育事业得到新发展，井冈山职业技术学院获省政府批复，中小学教育网点布局进一步优化，新吉安一中、新白鹭洲中学、吉安师范附属学校、特殊教育学校等四所学校和市中心医院城南分院向新区布点启动建设，全市完成189所中小学共20余万平方米校舍新建和改造任务。科技创新取得突破，全市获批64项国家和省级科技项目，6个科技项目获全省科技进步奖，获奖数列全省设区市之首；天人集团“松材线虫防治技术”获国家科技进步二等奖，为我市建国以来科技最高奖项。井冈山农业科技园即将获批国家级农业科技园。文体事业不断繁荣，全市53个乡镇综合文化站建设基本完成，611家农家书屋建成开放，农村文化“三项活动”深入开展，涌现了大型采茶戏《角色》等一批文艺创作精品，4幅作品入选全国美展，入选作品数量全省第一。市体育馆工程竣工使用，市第三届运动会成功举办。广播电视事业有新的进展，专题片《解放吉安》获全国纪念新中国成立六十周年突出贡献奖。卫生事业持续进步，公共卫生体系建设有效加强，甲型流感等疫情得到有效防控；市中心医院获全国医药卫生系统先进集体、晋升国家三级甲等综合医院，保健大楼建成使用；“光明·微笑”工程超额完成省下达任务，荣获全省先进市。民宗、统计、档案工作成效显著，市政府被国务院评为全国民族团结进步模范集体和全国第二次经济普查先进集体，市档案馆晋升国家二级馆。人口计生工作采取有力措施取得实际效果，农村妇女“双学双比”扎实开展。邮政通讯取得新的成绩，吉安“无线城市”建设进程加快。国防动员、民兵预备役、征兵、双拥、人民防空、国家安全工作得到加强，审计监察、外事侨务、对台事务、海关、检验检疫、公积金、粮食、供销、决策咨询、质监药监、气象水文、新闻出版等工作迈出新步伐，妇女、儿童、老龄、残疾人等事业进一步发展。安全生产形势总体平稳，社会治安综合治理和信访维稳扎实有效，全市社会和谐稳定。

　　（八）大力改进政风行风，政府自身建设得到加强。扎实开展深入学习实践科学发展观活动，广泛征求各方面意见，积极加强政府自身建设，建立完善了《市政府工作规则》、《政府重点工程投资评审管理暂行办法》、《市政府政务督查工作规则》等规章制度。建立重大事项向市委专报请示，向人大、政协通报，向社会新闻发布制度，完善市民听证咨询和民主评议制度，推进了政府决策民主化、科学化。全年共办理人大代表建议167件、政协提案225件，满意和基本满意率达100%。加强政务信息公开工作，全年公布政务信息11万条。加强“四中心一平台”惩防体系建设，进一步规范了行政服务中心和公共资源交易中心管理，让行政权力在阳光下运行。深入开展工程建设领域突出问题专项治理，强化政府投资工程监管，市投资评审办审核项目145批次7.42亿元，核减1.03亿元，核减率达13.5%。完善市长直通电话、市长信箱、市长手机、行政投诉、行风热线等平台建设和办理制度，全年受理群众来电来信19613件，办结率98%。扎实开展“机关效能年”活动，深入推进行政审批“两集中两到位”改革，精简市级行政许可项目217项、市级投资审批项目50项，精简率分别为52.3%和56.4%、办理时限压缩38%，发展环境进一步优化。出台并严格执行《关于压缩行政成本的规定》，机关厉行节约取得明显成效，行政会议、公车购置运行、公务接待等经费下降17%。

　　回顾一年来的工作，我们在市委的正确领导下，主要把握了以下几点：一是始终坚持主攻项目、决战“两区”。坚持以重大项目为主抓手、以园区和城区为主战场，紧抓项目建设不动摇，咬住扩大投资不放松，用一个个大项目来推动“两区”发展，有力地推进了新型工业化和新型城镇化进程，有效带动了全市经济社会发展。实践充分证明，市委确立的“主攻项目、决战两区”发展战略，完全符合吉安实际，抓住了吉安发展的主要矛盾，必须一以贯之地坚持下去。二是始终坚持改革创新、攻坚破难。针对发展的瓶颈制约，全市上下在困难面前不退缩，坚持以改革创新的办法破解难题，随着融资、拆迁、国企改革等难关的有效突破，一批想干而无力干的事项得到了落实，一些想推而推不动的项目得到了推进。实践证明，只要我们始终保持改革创新、攻坚破难的勇气，不断解放思想、善于学习，就一定能有效破解发展中的各种矛盾和问题，取得一个又一个胜利。三是始终坚持着力当前、统筹长远。一年来，我们坚持统筹兼顾的发展理念，既立足当前，注重抓好现阶段重点工作，全力解决当前保增长、保民生、保稳定等最紧迫、最现实的问题，又着眼长远，谋划和推进了一批事关全局的战略性工作，狠抓了一批产业项目的培植和专项规划、产业规划的编制完善，研究出台了一系列管长远的政策措施，为吉安科学发展打下坚实基础。四是始终坚持埋头苦干、不事张扬。一年来，各地各部门以深入学习实践科学发展观活动为契机，进一步加强思想和作风建设，始终保持“静悄悄急行军”的良好态势。各县市区开拓进取、埋头苦干、危中求进，县域经济快速发展；各市直部门善谋实干、主动履职、积极作为，各行各业捷报频传。全市上下到处呈现齐心协力、昂扬向上、扎实苦干、奋勇争先的良好氛围，这既是一年来各项工作取得突出成绩的重要保证，也是我们今后更好更快发展的宝贵经验。

　　各位代表，刚刚过去的一年，成绩来之不易，充分说明我们应对危机的措施是积极务实有效的，达成了保增长保民生保稳定的目标任务。这是党中央、国务院关心关爱的结果，是省委、省政府和市委正确领导的结果，是全市上下合力共为、团结奋斗，企业迎难而上，社会各界支持帮助的结果。回顾一年来的工作，我们尤其感念市人大和人大代表的依法监督与大力支持，感念市政协和政协委员的民主监督与建言献策，感念各民主党派和各界人士的热情参与和相助。在此，我代表市人民政府，向各位人大代表、政协委员，向奋斗在各条战线的全市广大干部群众，向离退休老同志，向各民主党派、工商联、无党派人士、各人民团体，向驻吉人民解放军和武警官兵、公安干警，向中央和省驻吉单位，向所有关心、支持和参与吉安建设的海内外朋友，表示衷心的感谢并致以崇高的敬意！

　　各位代表，刚刚过去的一年，我们也深深感到吉安经济社会发展中还存在不少困难和问题：一是欠发达的市情并未根本改变，经济总量和财力偏小，优势骨干企业还不多，重特大型项目还没有取得突破，综合经济实力依然不强。二是城镇化水平仍然偏低，城乡二元结构较为突出，中心城区功能不够完善，人气、商气不旺，以消费为基础的经济增长内生动力明显不足。三是农村基础设施仍然薄弱，农技推广等现代农业服务体系还不完善，农业产业化水平和农业经济效益还不高，农民持续增收的压力仍然较大。四是社会保障的能力和水平依然不高，尤其是失地农民保障政策及措施还不完备，改善民生和解决群众合理诉求的任务还十分艰巨。五是政府职能转变还不能完全适应发展的需要，改革创新和破解难题的能力离党和人民群众的要求还有距离，政务环境和行政效能还需要进一步优化和提升。对此，我们将始终保持清醒头脑，采取更加有力措施，认真加以解决。

　　二、2010年政府工作目标和主要任务

　　2010年是“十一五”规划的收关之年，也是吉安撤地设市十周年，更是吉安赶超发展极为重要的一年。从发展形势看，当前我国经济稳步回升的态势已经确立，世界经济也出现了回暖迹象，为我们加快发展提供了良好的外部环境；国家继续实行积极的财政政策和适度宽松的货币政策，为我市主攻项目、扩大投入提供了良机；国家强力推进区域经济发展，尤其是我省《鄱阳湖生态经济区规划》上升为国家战略，为我们决战“两区”、主动承接辐射带动，加快融入新一轮产业调整布局提供了广阔空间；我市近年来实施的一大批重大基础设施和产业项目，为我们加速崛起奠定了良好基础；更为重要的是，我市的发展战略得到全市上下高度认同，各级干部经过大量的探索实践，推动科学发展、破解发展难题的本领不断增强，全市上下合力共为、苦干实干的氛围已经形成，必将对吉安的进位赶超、跨越发展产生巨大的推动作用。

　　我们也要清醒地看到，国际金融危机的影响在短期内还很难消除，我国经济复苏的基础还不稳固，保持经济平稳较快发展的难度还很大；尤其在这一轮经济波动中，全省各地抢抓机遇、竞相发展的势头更加强劲，赶超发展的压力更趋激烈。我们务必充分认识发展中的困难和问题，进一步增强加快发展的危机感、紧迫感和责任感，倍加珍惜来之不易的良好发展局面，坚持好思路，完善好举措，保持好势头，全力以赴促进经济社会更好更快发展，千方百计加快进位赶超，努力开创吉安跨越发展新局面。

　　今年，政府工作的总体要求是：以科学发展观为指导，以“三保一弘扬”为统领，积极策应鄱阳湖生态经济区发展战略，坚持“两个略高于”发展不动摇，坚持主攻项目、决战“两区”不动摇，把保增长、扩内需、调结构、促改革、惠民生结合起来，更加注重统筹城乡发展，更加注重统筹经济社会发展，更加注重统筹当前与长远发展，更加注重统筹改革、发展和稳定，推动全市经济社会进位赶超、跨越发展。按照上述要求，综合考虑各方面因素，2010年全市经济社会发展主要预期目标是：生产总值增长12%，其中规模工业增加值增长20%；财政总收入增长16%；固定资产投资增长35%；社会消费品零售总额增长18%；外贸出口增长10%；实际引进市外资金增长10%，实际利用外资增长10%；城镇居民人均可支配收入增长9%，农民人均纯收入增长8%；居民消费价格总水平涨幅控制在3%以内；城镇登记失业率控制在4%以内；人口自然增长率控制在7.8‰以内；单位GDP综合能耗下降2.2%，化学需氧量和二氧化硫排放量分别下降1.4%和5.6%。为实现上述目标，主要抓好以下工作：

　　（一）以吉泰工业走廊为核心，推动“决战园区”再上新水平。坚定不移地实施“决战园区”战略，以建设吉泰工业走廊为核心、以井开区为龙头，推动工业经济跨越发展。

　　加快吉泰工业走廊建设。集中力量抓好走廊规划的研究论证。加强走廊区域基础设施和服务平台建设，加快推进105国道“二改一”工程，推进电信和公交一体化，确保上半年开通中心城区至泰和、吉水公交线路；抓紧做好井开区设立出口加工区和升级为国家级经济技术开发区工作，全面启动吉安现代物流园和电子元器件专业市场建设；进一步细化产业规划及政策扶持措施，引导走廊内“三县三区”重点围绕电子信息、生物医药两大产业，加大产业招商及配套企业引进力度，努力将吉泰工业走廊打造成为全国重要的电子信息产业基地。推进园区大投入、大建设、大发展。继续举全市之力推动井开区加快发展，重点围绕井开区升级，完成46.5平方公里规划编制，抓好水、电、路、气及中央商务区等专项规划，争取深圳南山区支持，加快深圳产业园建设。启动跨铁路5000亩土地的征地拆迁，实施总投入10亿元以上的路网建设等基础工程，加快园区商场、学校、医院、住宅小区等生活性配套设施，以及　　 物流园、标准厂房等生产性配套设施建设，确保年内井开区规模工业实现主营收入150亿元、财政收入增长30%以上。同时，积极引导各县区工业园进一步完善园区规划，加快完善基础设施及配套服务，加强园区土地节约集约利用和闲置土地清理，力争每个园区重点培育1-2个主导产业、5-10户重点企业，打造一批主业突出、特色鲜明、配套完善的产业园区。

　　实施“双百双十”工程，促进产业优化升级。围绕2011年工业产值过千亿目标，在全市启动“双百双十”工程，打造百亿优势产业和百亿工业园区，培育收入十亿企业和市场，不断做大经济规模，调优产业结构。全力壮大支柱产业，加大对电子信息、医药化工、食品加工等六大支柱产业的扶持力度，拓展产业规模、延伸产业链条、提升产业层次；同时，坚持引进高新技术产业，积极改造传统产业，加快培育光伏材料、半导体照明等战略性新兴产业，不断构筑产业新优势。积极实施品牌战略，争创驰名、著名商标。全力做强骨干企业，继续落实保企业的各项政策措施，加强对重点企业的调度帮扶，协助一批骨干企业制订三年发展目标规划，促其尽快做大做强，力争全年主营业务收入过亿元企业达到160户以上。全力推进重大项目，组织开展工业、商贸重点项目建设年活动，年内重点抓好中泰电子、麦德风能等129个增长点项目的达产达标，杰彼新能源、满坤科技、安昌铝业等150个在建亿元以上项目的竣工投产，中信华电子等一批工业和商贸签约项目的开工建设，切实提高项目的资金到位率、竣工投产率和税收贡献率。

　　（二）以中心城区和县城为主战场，推动“决战城区”再上新台阶。积极策应国家重点发展中小城市和小城镇的政策机遇，继续以中心城区和县市城区为主战场，紧扣规划、建设、经营、管理四个方面，全力破解投入、征地、拆迁、监管四大难题，进一步加大“决战城区”力度。

　　努力完善提升城镇规划。启动编制《吉安市中心城市群总体规划》，配套推进吉泰走廊城镇建设，加快走廊沿线人口向城镇聚集，努力构建“三区三县”各具特色城镇群，逐步将吉泰工业走廊打造成推进新型工业化、新型城镇化和城乡一体化、体制机制创新试验区。同时，抓紧编制中心城区和各县市控制性详规，补充完善供电、供水、供气、公交等基础设施，以及商业网点、学校、医院等公共服务专项规划，不断完善规划体系。

　　高标准推进城镇建设。按照基础设施优先、社会事业优先、民生需求优先、聚集人气优先、绿色环保优先原则，以更高的标准、更大的投入，加快城镇建设步伐。中心城区全力推进城南新区和滨江新区建设，继续完善路网等市政基础工程，加速启动一批社会事业、公共服务、商贸市场、商业楼宇、房产开发等功能性、经营性项目。确保建成航校新机场、市民中心公园、庐陵文化生态公园一期、环湖路等项目，开工建设市博物馆与城市展示馆、吉州大道南延二期等项目，抓紧启动新井冈山大桥等工程。在老城区继续实施以白改黑、管网改造、街景改造、棚户区改造四位一体的改造工程，完成青原大道、吉州大道、跃进路、华能大道等城市道路和污水管网改造；启动马铺前、宝华楼、樟脑厂、砖瓦厂等棚户区改造77.7万平方米；抓好青原大道、吉州大道、韶山路、鹭洲路街景整治，努力营造优美宜居环境。建设一批公厕、垃圾中转站等便民项目，努力完善城区功能。在推进中心城区建设中，继续加大筹资力度，组织好新一轮征地拆迁，为项目建设提供有力保障。县城建设按照强化功能、提升品位的要求，注重推进路网工程、垃圾处理、公园绿地等市政基础项目，合理规划建设图书馆、文化馆、体育场馆及学校、医院、商贸市场等公共设施，不断增强县城集聚产业、吸引人气、聚集商气的承载力。小城镇建设着力抓好列入省重点的14个镇的规划建设，努力探索实践，突出解决规划标准低、建设档次低、市政配套差、为农服务业弱等问题，努力打造成全市小城镇建设的示范样板。其他乡镇以乡镇规范化建设为抓手，加强乡镇规划，加快小城镇供水、路网改造和环境整治，不断增强小城镇为农服务的能力。

　　全面提升城管水平。继续深入推进“三城同创”，组织实施中心城区环卫体制改革，建立健全清扫、保洁、监督三支队伍，推进环卫保洁作业重心下移，实现市场化、全覆盖。创新城市管理手段，探索建立负责任、全天候城市监督管理以及数字城管，提升城管实时监测和动态控制能力。注重物业管理市场培育与监管，提升小区物业管理水平。

　　努力扩张城镇规模。实施城镇人口倍增计划，抓紧研究制订中心城区和县市城镇人口十年倍增实施办法，出台鼓励外来人口落户中心城区及县市城镇的激励政策。全面放宽城镇户籍限制，对外来落户人口给予就业、就学、就医、就居同等政策，统一纳入城镇创业培训、就业指导和社会保障体系，吸纳更多农业人口和高素质人才在城镇就业落户，确保全市城镇化率提高1.6个百分点。

　　（三）继续抓好“三农”工作，夯实经济发展基础。坚持把“三农”工作摆在重中之重，加强农业基础建设，加快农业产业化进程，促进农业增效、农民增收、农村和谐。

　　加快农业产业化进程。全面落实国家惠农政策，稳定粮食生产，调优粮食品种结构，重点抓好粮食高产创建万亩示范片和“百万超级稻”工程建设；继续壮大草食畜禽、特种水产等农业主导产业，力争肉牛出栏增长8%、特种水产品增长10%；突出培育油茶、蜜柚等新兴富民产业，组织实施好100万亩高产油茶林基地建设项目，推进井冈蜜柚产业“百千万”工程；注重规划发展城郊蔬菜基地，努力培育一批蔬菜种植专业户、专业村，补充调剂市区蔬菜供应。继续以农业产业化“五大行动”为抓手，着力引进、扶持一批带动力强的龙头企业，培育支持一批农民专业合作社，推进优势农产品生产标准化、经营规模化、产品高效化，带动农民持续增收。

　　扎实推进新农村建设。市财政配套安排1358万元支持新农村建设，重点抓好823个试点村和第二轮综合示范点创建工作。以中心城区和县城城郊及工业园区周边村为重点，打造城郊经济型新农村建设试点圈；以交通干线沿线为重点，打造连点成线的新农村建设示范带；以乡镇驻地村、集镇和旅游景区为重点，打造整村推进型新农村建设试点片。加紧完善村庄规划，继续抓好“三清三改”、“五通一气”、“三绿化一处理”工程，探索建立“户集、村收、镇运、县处理”的乡村垃圾无害化集中处理运行机制，切实改善村庄环境。大力推进农村社区建设，力争每个重点乡镇都有一个新农村社区示范点。

　　完善农业服务体系。健全农村公共信息服务网络，建立市本级突发公共事件应急流动气象台，完善吉安市防灾减灾预警主叫服务系统。高度关注基层农技推广体系“线断、网破、人散”状况，探索采取“三定向”方式培养或聘用农技人员充实基层。切实加强产前、产中、产后服务。大力推广农业机械化，力争耕种收综合机械化率达到30%以上。充分发挥供销社在农村现代流通的作用，进一步完善县市区农资物流配送中心。

　　（四）坚持推动项目建设，强力扩大有效投入。坚持把抓项目、扩投资作为经济发展的核心，继续保持主攻项目的强劲态势，全力推进一批市重点项目，以大项目带动大投入，大投入拉动大发展。

　　千方百计争取项目。认真把握国家投资的重点和取向，围绕国家确定的投资领域，抓紧重大项目包装、论证，做好政策、项目、资金对接落实，力争在国家投资的总盘子中占有更大份额。同时，着眼于对接融入“十二五”规划和鄱阳湖生态经济区，重点围绕我市“四大基地”优势主导产业、农业基础设施、旅游、生态环保、民生需求，谋划一批重大项目，加强项目生成储备，争取挤进国家和省中长期发展规划，为吉安经济社会发展增添后劲。

　　全力以赴推进项目建设。重新筛选一批事关经济社会长远发展的重大项目，完善项目推进和监管机制，不断加快项目建设步伐。交通方面：重点推进衡茶吉铁路及文茶联络线工程、泉南高速石城至吉安段工程、吉安至莲花高速建设，抓好京九电气化改造，力促开工建设昌吉赣铁路客运专线和抚州至吉安、厦坪至睦村高速公路，大力推进衡茶吉东延吉安至建宁至武夷山铁路、井冈山至赣州铁路、105国道环城和绕城高速等项目前期工作，启动实施井冈山机场、吉安火车站改扩建工程，抓好迎国检线路105、319国道、抚八线、吉福路等路面大中修工程及青原至东固公路改造工程。完成通村水泥路1200公里、农村渡改桥22座，全面实现村村通公路，全面完成渡改桥任务。能源方面：重点加快峡江水利枢纽、石虎塘航电枢纽等工程建设，推进万安核电工程和井冈山梯级电站、华能井冈山电厂三期工程前期工作；抓好以5座220千伏、6座110千伏输变电站为骨干，吉泰走廊电网、井冈山市电网、井开区电网建设改造为重点，总投资14亿元的电网改造，打造一流电力网络。农林水利方面：着力抓好吉安市大型商品粮生产基地续建项目，全面完成中央规划内的83座病险水库除险加固任务，抓好永丰、吉水、安福3个小型农田水利重点县建设，加快建设中心城区堤防工程。社会事业方面：确保建成文化艺术中心等项目，启动建设广电传媒中心、奥林匹克公园等项目，抓紧实施中小学校舍安全工程和农村卫生服务体系建设工程，加快中心城区4所新校区、1所新医院建设步伐。

　　多元融资保障项目。完善政府七大投融资公司的经营管理制度，健全投融资公司“借、管、用、还”机制，促进国有资产保值增值，提升可持续投融资发展能力。加快信用体系建设，优化金融生态环境，防范化解金融风险。创新政银企合作方式，积极引进境内外金融机构在我市驻点，争取金融机构更多的信贷支持。积极与央企开展BT合作，加大争取世行、亚行贷款力度。加强企业上市的政策扶持与业务辅导，加快天人生态、普正药业等企业上市融资步伐。进一步鼓励和扩大民间资本进入市政公用事业、基础设施、社会事业和现代服务业等领域。

　　（五）加快发展现代服务业，全力扩大消费需求。抓住国家扩大内需特别是增加居民消费需求的机遇，适应产业发展需求，大力发展现代服务业，不断提升服务业对经济增长的贡献率。

　　继续做强旅游产业。加快景区景点开发，按照建设旅游大市的要求，重点抓好井冈山茨坪、茅坪、领袖峰等景点改造工程，推进武功山金顶景区整体开发等项目建设，加大东井冈、万安湖、武功湖等旅游资源开发力度，挖掘开发渼陂、钓源为代表的庐陵文化和青原山为代表的宗教文化旅游产品，全力构建以井冈山为龙头的大井冈旅游经济圈。完善旅游宾招服务设施，中心城区和井冈山力争启动建设3个五星级酒店，其他县区启动建设一批三星级以上酒店，努力实现游在吉安、留在吉安的目标。强化旅游宣传推介，切实加大旅游宣传推介力度，继续举办井冈山红色旅游文化节，着力提升吉安“横江葡萄节”、泰和“乌鸡节”、新干“箱包节”等节庆活动水平，精心打造一批精品旅游线路，积极研发推出一批旅游商品，提升吉安旅游的吸引力、影响力。整合旅游经营性优质资产，推动井冈山旅游上市，加强山上山下资源互动共享，引进大型旅游龙头企业参与开发建设，促进全市旅游总人数和总收入再上新台阶。

　　大力发展商贸物流业。以开拓城乡市场、服务生产生活、扩大居民消费为重点，加快构建安全、便捷、畅通、高效的商贸流通体系。围绕扩大城市消费，科学规划商业网点，重视建设物流园区，大力发展物流配送、连锁经营等新型业态，引进中端以上商家入驻吉安，加快推进城南钢材市场、九天商厦、农产品物流中心、城北汽车市场、广场高端商城等一批市场建设项目，提升城区商业水平。围绕促进农村消费，继续推进“万村千乡市场工程”和乡镇商贸“六个一”工程，全面落实家电下乡、农机下乡、汽车摩托车下乡政策，拉动农村消费市场。积极培育新兴服务业。大力发展文化产业，制定出台文化产业发展规划和扶持政策，鼓励发展演艺、娱乐、影视等文化产业，满足市民精神文化需求。大力发展会展经济，加快梨坪国际会议中心建设，适时启动中心城区会展中心项目，积极组织吸引一批会展商来我市举办会展，推动会展业加快发展。积极培育总部经济、楼宇经济，高标准规划总部经济发展区，研究出台促进总部经济发展的扶持政策，吸引一批企业总部或区域总部落户我市。同时，支持TD和3G移动通信系统的建设运营，加快推进广电网、电信网和互联网三网融合。

　　（六）深化改革开放，激发经济发展动力和活力。坚定不移地推进改革开放，加快重要领域和关键环节改革步伐，拓展开放广度和深度，为全市加速发展、进位赶超提供强大动力。纵深推进各项改革。继续推进国企改革。在做好国有工业企业改制后续工作的同时，着力抓好商贸、粮食、建设等非工国有企业的改制工作，确保企业改制顺利完成。有序推进政府机构改革。以转变职能为核心，全面完成市县机构改革任务；以完善乡镇便民服务体系为重点，进一步深化乡镇机构改革，力争３年内基本完成；以健全人事、职称和分配“三项制度”为目标，大力推进事业单位分类改革。稳妥推进医药卫生体制改革。加强基本医疗保障制度建设，将国有关、改、破和困难企业职工纳入基本医疗保障，确保城镇职工医保、居民医保实现全覆盖；逐步提高城镇职工医保、城镇居民医保和新农合政策范围内的门诊及住院费用报销比例，提升基本医疗保障水平；在全市所有中心卫生院实施国家基本药物制度试点，有效缓解群众“看病难、看病贵”问题。积极推进文化体制改革。制定文化体制改革方案和配套措施，加快文化事业单位改制步伐，大力培育文化市场主体，推动文化事业繁荣发展。此外，还要积极探索社会管理创新。

　　全面扩大对外开放。策应国际经济格局和产业分工新一轮调整，进一步提升对外开放的能力，拓展对外开放领域，依托大开放，促进大发展。进一步加大招商安商力度。继续配强专业招商队伍，加大产业招商力度，重点围绕电子信息、医药化工、先进制造、新能源等产业，积极引进一批投资规模大、科技含量高、经济效益好、环境污染少的大项目、好项目；积极靠大联强，加强与央企对接，争取引进一批央企落户。同时，着眼完善产业配套，围绕主导产业、支柱企业，着力引进上游供应产品、下游终端产品制造企业，重点抓紧引进电子信息产业急需配套的精密机械加工企业，鼓励已落户的核心龙头企业及时引进相关配套企业，推进产业链条式延伸，实现企业集群式发展。整合培训资源，大力实施“蓝领培训”计划，推行人力资源“订单培训”，为项目入驻和企业发展解决技术工人不足问题。进一步扩大外贸出口。充分运用出口退税、外贸发展资金等政策措施，加大对出口企业的扶持力度；支持企业转变外贸发展方式，扩大机电、高新技术和自主品牌　　产品出口比重，确保出口总额持续强劲增长。

　　加快发展民营经济。充分落实激活创业的政策措施，加大对民营经济的扶持力度，保护民间投资合法权益。大力培育创业文化，引导鼓励大中专毕业生、转业退伍军人及下岗工人自主创业，农村基层干部群众致富创业，外出务工人员返乡创业，科技人员兴办实体创业，努力营造全民创业的良好环境，推进民营经济发展扩规模、提速度、上水平。

　　（七）着力建养生态，切实增强资源环境承载能力。坚持把绿色生态放在重要位置，大力发展低碳经济，推进节能减排，创建生态园林城市，努力打造资源节约型、环境友好型社会。

　　积极策应鄱阳湖生态经济区建设。在功能区划、发展规划、产业培植和生态建设上，积极对接鄱阳湖生态经济区建设，突出发展低碳与生态经济，努力打造以生态农业、绿色生态家园、绿色产品、新型工业、新型能源、现代服务业和生态工业园为支撑的“低碳吉安”，跟进鄱阳湖生态经济区的发展步伐。

　　大力推进节能减排。鼓励发展循环经济，大力提升废物资源化和再生资源回收利用水平，突出抓好火力发电、冶炼等重点耗能行业的节能降耗，加快淘汰落后生产能力，加大公共机构节能力度，全面完成省政府下达的节能任务。突出抓好造纸、盐化、电镀、金属冶炼等重点行业和赣江等重要水系两岸企业的污染防治，切实提高企业环保准入门槛，特别是城市上游园区、水资源地、城市用水取水口地区的环保标准，加大环保执法力度，确保全市水环境质量保持国家Ⅲ类以上标准，饮用水源达标率100%，使吉安的天更蓝、水更清。

　　抓好城乡园林绿化。围绕构建生态园林城市，继续加大中心城区和城镇园林绿化力度，将森林引进城市，大幅增加城镇绿化面积，提升绿化品位。积极开展赣江吉安段生态示范带建设工作，继续抓好环境优美乡镇及生态示范村创建活动。打好造林绿化“一大四小”工程建设三年攻坚战，抓好交通干线可视范围内、交接线绿化和楠木上山、樟树护村、油茶富民、新农村绿化样板工程，使吉安的山更青、地更绿。

　　（八）全力改善民生，促进社会和谐稳定。

　　始终把改善民生、促进民富放在首位，让广大群众在发展中得到更多实惠，不断增强群众的幸福感，提高社会的和谐度。

　　千方百计推进民生工程。抓好就业再就业工作，全面落实积极的就业政策，整合“金蓝领工程”、“阳光工程”、“雨露计划”等各类资金，搞好就业创业培训，重点解决好返乡农民工、高校毕业生等群体的就业问题，力争政府公益性购岗6000个以上，确保全年新增城镇就业4.5万人以上。提升社会保障水平，加大社保扩面征缴工作力度，重点抓好新型农村养老保险试点和企业职工养老保险工作，建立完善失地农民保障机制。实施好“蓝天计划”，启动吉安儿童福利院和老年公寓建设。完善社会救助体系，继续提高补助水平，将中心城区城市低保标准提高到275元、农村低保标准提高到110元，将医疗救助最高封顶线提高至３万元。改善群众生产生活条件，合理引导房地产业，加大普通商品住房供应，推进房地产健康发展；继续抓好保障性住房建设，开展廉租房共有产权管理试点，全市建成经济适用房1002套、廉租房5524套；组织实施好农村危房改造工程；继续实施移民扶贫、农村安全饮水、农村沼气等工程，重视抓好峡江水利枢纽移民工作，完成１万名移民扶贫搬迁任务，解决21万农村人口安全饮水问题，新增农村沼气用户1.6万户。优先发展城市公交，抓紧做好中心城区公交规划，开工建设公交总站，筹建城北汽车站，启动长途汽车站迁建工程；合理布局公交网络，科学设定站点，完善公交站牌，新辟和延长公交线路，改善广大市民出行条件。

　　千方百计发展社会事业。按照全面协调可持续发展的要求，在加快推进经济建设的同时，大力发展各项社会事业。促进教育均衡发展，加强学前和义务教育管理，切实解决好困难家庭子女上学问题；加大中小学标准化建设力度，每个县市重点抓好一所中学的标准化建设，不断改善办学条件，提升教育质量；抓紧编制全市职业技术教育规划，加快推进井冈山职业技术学院建设，努力促进职业教育发展。推进科技体制创新，重视引进人才，构筑人才高地。大力实施科技创新“六个一”工程，主攻一批高新技术产业，培育一批创新型企业，申报一批国家和省重大科技项目，建立一批重点研发平台，办好一批技术开发与产业化特色基地，组建一批优势科技创新团队。完善公共卫生体系，健全农村三级医疗卫生体系，抓紧筹建市医疗急救指挥中心，继续实施“光明·微笑”工程，加强甲型流感等重点传染病的防控工作，加强社区卫生机构和医院规划布点建设。全面繁荣文体事业，继续抓好广播电视村村通、农村文化三项活动、乡镇综合文化站建设、信息资源共享、非物质文化遗产保护等文化广电惠民工程，新增广播电视“村村通”自然村640个，组织开展系列文化艺术活动；推进社区体育健身场所和幼儿玩乐场所建设，广泛开展全民健身活动，积极备战全省第十三届运动会。切实抓好计生工作，以“四项手术”为突破口，稳定低生育水平，加大出生人口性别比综合治理力度，确保完成省政府下达的人口控制计划。同时，重视全民国防教育，扎实推进国防后备力量建设，抓好统计、物价、人防、民族宗教、外事侨务、涉台事务、档案方志等工作，促进各项事业全面进步。

　　千方百计维护社会稳定。高度重视安全生产，严格落实安全生产责任制，进一步强化重点行业、重点领域、重点场所的安全监管，坚决杜绝重特大安全事故的发生；切实加强森林防火和消防安全工作，坚决遏制重特大火灾的发生。加强食品药品质量监管，提高食品药品安全综合监管能力，杜绝重大食品药品安全事故发生。认真做好信访工作，严格落实信访工作责任制，深入做好矛盾纠纷排查调处，进一步加强和改进新形势下的群众工作和信访工作；坚持和完善领导干部大接访制度，切实把矛盾解决在基层，消除在萌芽状态。办好市长手机、市长信箱、市长直通电话，畅通诉求渠道。强化社会治安综合治理，提高社会治安防控能力，推进“天网”工程建设，加大严打整治力度；加强应急管理工作，健全社会预警体系和应急机制，不断提高应对各类突发事件和保障公共安全的能力，确保社会平安稳定。

　　三、加强政府自身建设

　　完成今年的目标任务，实现吉安进位赶超、跨越发展，对政府工作提出了新的更高要求。我们要着力加强政府自身建设，努力提高、改进政府工作能力和工作作风，努力建设“为民、务实、高效、廉洁”政府。

　　（一）牢记宗旨，努力建设情系百姓的为民政府。坚持以人为本，执政为民。倡导各级政府从群众反映最强烈的问题抓起，从群众最企盼的事情做起，从柴米油盐、衣食住行、入学就业、求医问药、助老托幼、扶残助弱等方面入手，尽心竭力办好事、办实事。特别是在推进经济发展中，更多地关注民计民生；在加快城市建设中，更多地考虑利民为民；在强化社会管理时，更多地体现人文关怀。对事关经济社会发展全局的重大事项，深入调查研究，广泛征询群众意见，做到问政于民；对群众日常生活和切身利益密切相关的社会事项，实行社会公示和听政制度，做到听政于民；继续抓好政务信息公开和新闻发布，办好“政风行风热线”，保障公民的知情权、表达权、参与权和监督权，做到议政于民。

　　（二）善谋实干，努力建设创新求进的务实政府。着眼于破解难题、突破瓶颈，加强现代金融知识、经济政策、创新手段和先进经验等方　　 面的学习研究，进一步解放思想，锐意改革，努力探索发展新理念、新方法。倡导精细、务实的工作作风，主动深入条件艰苦、矛盾集中的一线了解情况，更多地现场办公解决问题、推动工作，坚决杜绝作风漂浮、脱离群众、麻木不仁等现象。进一步精简会议、文电，改进会风、文风，真正把心思用在干事业上，把精力放到抓发展上。进一步强化目标责任考核，以目标倒逼进度，以督查倒逼落实，确保市委的决策部署落到实处，努力在全市形成察实情、干实事、求实效、狠抓落实的浓厚氛围。

　　（三）提升效能，努力建设勤政快捷的高效政府。积极转变政府职能，将各级行政机关的工作重点转移到为市场主体服务和创优发展环境上来，着力解决影响行政效能的突出问题。扎实开展“创业服务年”活动，全面落实《关于创优发展环境的实施意见》，为企业发展和全民创业营造宽松环境。深化行政审批制度改革，年内建成网上审批及电子监察系统，实现审批提速、服务提效。完善行政服务中心和乡镇便民服务中心建设，为企业、客商、群众提供更加便捷的办事服务平台。加强部门协作和效能问责，加快各项工作及各重点项目的推进，坚决克服推诿扯皮、各自为政现象，坚决查处损害经济发展环境的典型案件，倾力打造一流的发展环境。

　　(四)从严治政，努力建设风清气正的廉洁政府。把依法行政贯穿政府决策、执行、监督的全过程，严格按照法定权限和程序履行职责。健全公众参与、专家论证和政府决策相结合的科学民主决策机制，规范政府重大决策行为。认真接受市人大的法律监督、工作监督和市政协的民主监督，全力办理人大代表建议和政协提案；主动听取各民主党派、工商联、无党派人士和各人民团体意见，自觉接受新闻媒体和社会公众监督。认真落实政府部门党风廉政建设责任制，坚持重大事项报告制度、行政问责制度，继续完善“四中心一平台”建设，规范行政权力运行，建立健全源头预防和治理腐败体系。切实加强对项目资金及工程建设的监督，探索推行政府投资项目代建制，确保重点工程成为优质工程、廉洁工程。坚持艰苦奋斗、厉行节约，倡导勤俭办一切事情，节约办一切活动，将有限财力更多投向民生社会事业。加大行政监察和审计监督力度，严肃查处违法违纪行为，坚决纠正损害群众利益的不正之风。

　　各位代表，吉安作为革命老区，曾为中国革命做出过巨大贡献和牺牲，在这块土地上工作的各级干部身负加快地方发展、加快百姓致富的沉甸甸责任。让我们在市委的坚强领导下，大力弘扬井冈山精神，不辜负党的重托、人民的期盼，肩负起任务使命，振奋精神、顽强拼搏，为建设科学发展新吉安、绿色发展新吉安、和谐发展新吉安而努力奋斗！
